# Phage Genetic Engineering Using CRISPR–Cas Systems

**DOI:** 10.3390/v10060335

**Published:** 2018-06-19

**Authors:** Asma Hatoum-Aslan

**Affiliations:** Department of Biological Sciences, University of Alabama, Tuscaloosa, AL 35487, USA; ahatoum@ua.edu; Tel.: +1-205-348-4097

**Keywords:** CRISPR–Cas, bacteriophage, genome editing, phage genetics

## Abstract

Since their discovery over a decade ago, the class of prokaryotic immune systems known as CRISPR–Cas have afforded a suite of genetic tools that have revolutionized research in model organisms spanning all domains of life. CRISPR-mediated tools have also emerged for the natural targets of CRISPR–Cas immunity, the viruses that specifically infect bacteria, or phages. Despite their status as the most abundant biological entities on the planet, the majority of phage genes have unassigned functions. This reality underscores the need for robust genetic tools to study them. Recent reports have demonstrated that CRISPR–Cas systems, specifically the three major types (I, II, and III), can be harnessed to genetically engineer phages that infect diverse hosts. Here, the mechanisms of each of these systems, specific strategies used, and phage editing efficacies will be reviewed. Due to the relatively wide distribution of CRISPR–Cas systems across bacteria and archaea, it is anticipated that these immune systems will provide generally applicable tools that will advance the mechanistic understanding of prokaryotic viruses and accelerate the development of novel technologies based on these ubiquitous organisms.

## 1. Introduction

Bacteriophages, or phages for short, are viruses that infect bacteria. Phages are vast in number, genetically diverse, and due to their high proportion of genes with unknown functions, have been aptly described as the “dark matter” of the biological universe [1]. With an estimated 10^31^ phage particles in the biosphere, they outnumber their bacterial hosts by a factor of 10 [2,3]. Phages play integral roles in the ecosystems within and around us as key drivers of bacterial evolution and major conduits of gene exchange [4,5,6,7,8,9]. A recent study showed that in addition to marine and terrestrial ecosystems, phages are also abundant in the atmosphere, traveling across continents on dust particles and debris, raining back down onto distant environments [10]. In a sense, phages are akin to a portal through which a vast reservoir of genetic material can gain transit through space and time. With as few as ten genes, a phage can build its structural proteins and execute a genetic program that leads to its own replication [11]; however, at the time of writing, the ~2200 complete phage genomes in the NCBI (National Center for Biotechnology Information) database encode an average of about 102 genes (Table S1). This observation begs the question—what are all the other genes doing?

Historically, a basic understanding of phage biology has been gleaned from research into a handful of phages that infect *Escherichia coli* [12,13,14]. Beyond providing fundamental insights into phage–host dynamics, early research using these model organisms has helped to establish the central dogma of molecular biology. Notable milestones include the revelation that heritable information is encoded in DNA, not proteins [15]; the discovery of the role of mRNA in protein synthesis [16]; and the first observation that mRNA is read three nucleotides at a time [17]. Along the way, phage research has also provided a plethora of tools, such as T4 DNA ligase and diverse restriction enzymes, which have since become vital staples in any laboratory that endeavors to employ molecular cloning techniques. Presently, in the wake of the recent characterization of a yoghurt bacterium’s anti-phage immune system, composed of a CRISPR (clustered regularly-interspaced short palindromic repeats) locus and Cas (CRISPR-associated) proteins [18], what began as basic phage research has led to the development of a suite of genetic tools that continue to revolutionize genetics and biomedical research [19]. With the advent of CRISPR–Cas genome editing technologies, research into the complex relationship between phages and their bacterial hosts has now come full-circle to provide the very tools by which a much deeper understanding of phage genetics is now possible.

## 2. Phage Genomes Abound with Genes of Unknown Function

Although the existence of phages has been known for over a century [20,21], functions for the majority of their genes remain uncharacterized. Even in one of the earliest molecular genetic models, *E. coli* phage T4 [22], 128 out of its 278 gene products (46%) remain annotated as “hypothetical protein” (GenBank accession NC_000866.4). The fraction of hypothetical genes is even higher when examining large phage collections: in a recent study, which offered an unprecedented look into the genomes of 627 bacteriophages from distal geographical locations that infect a single host, 75% of the collective phage genes could not be given functional assignments [23]. Indeed, the preponderance of hypothetical genes is also apparent in the complete phage genomes listed in the NCBI database (https://www.ncbi.nlm.nih.gov/genomes/GenomesGroup.cgi). According to our own analysis, the annotation “hypothetical” was given to approximately 63% of all gene products (Table S1). It is important to note that this number may be a conservative estimate, as a great many of the genomes do not contain functional predictions in their database entries. In view of the fact that next-generation sequencing technologies have made high-throughput phage sequencing more broadly-accessible with record turnaround times [24,25], it is anticipated that the number of complete phage genome sequences will grow at an accelerated pace, along with the number of unique hypothetical proteins within these collections. 

The problem of accurate characterization of the ever-increasing number of hypothetical proteins represents a serious bottleneck in phage research, owing, in part, to the lack of robust and generally applicable tools to study them. Temperate phages, which integrate into the host chromosome and remain dormant for generations, can feasibly be accessed and engineered using the same strategies developed for the bacterial host. However, lytic phages, which infect and kill their host within minutes to hours, remain intractable by most existing genetic engineering techniques [26]. Classical strategies that rely solely on homologous recombination between the phage genome and a donor DNA construct require time-consuming screening efforts to recover the desired mutant, due to low recombination rates and lack of selectable markers [27,28]. Alternatively, strategies that involve the transformation of bacterial hosts with synthetic phage genomes [29,30,31] are unsuitable for use in host bacteria that exhibit low/no competence. A recent study showed that this issue can be overcome in some Gram-positive organisms using cell wall-deficient hosts [32]; however, the general applicability of this approach remains unknown. Against the backdrop of all these techniques, distinct CRISPR–Cas systems in diverse bacteria have recently been used with great success to assist in phage genome engineering [28,33,34,35,36,37]. According to the CRISPRs web server, an online service that detects and lists the presence of CRISPR systems in sequenced prokaryotes [38], about 45% of bacteria and 87% of archaea are predicted to harbor one or more CRISPR–Cas systems. Therefore, the natural abundance of CRISPR–Cas systems in a broad range of hosts creates a unique opportunity to harness them as genetic tools to engineer the viruses that infect them.

## 3. Harnessing CRISPR–Cas for Phage Genome Engineering

**Figure 1:**
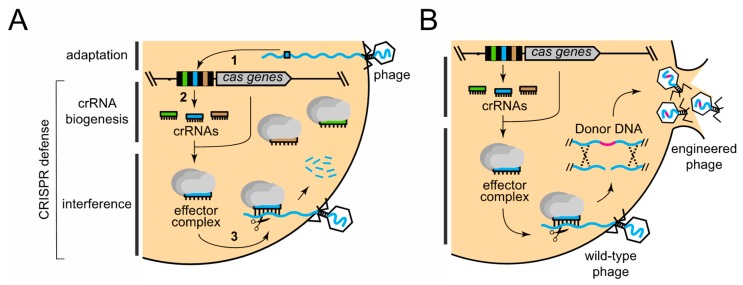
The CRISPR–Cas pathway and its application to phage genome editing. (**A**) The general three-step mechanism of CRISPR–Cas immunity consists of (1) adaptation; (2) crRNA biogenesis; and (3) interference. The latter two steps constitute CRISPR defense. Within the CRISPR locus, DNA repeats (black rectangles), spacers (colored rectangles), and *cas* genes (grey arrow) are shown. (**B**) The approach to using CRISPR defense for phage editing, wherein defense is used as a counter-selection mechanism to enrich for recombinant phages that have acquired mutations in the desired gene(s) from a donor DNA construct.

CRISPR–Cas systems are a class of adaptive immune systems that use small CRISPR RNAs (crRNAs) and Cas nucleases to detect and destroy foreign nucleic acids [18,39,40,41]. In general, CRISPR–Cas immunity occurs in three steps: (i) adaptation; (ii) crRNA biogenesis; and (iii) interference (Figure 1A). During adaptation, short (30–40 nucleotide) invader-derived sequences called “spacers” are captured and integrated into CRISPR loci in between partially palindromic DNA repeats of similar length. During crRNA biogenesis, the repeat–spacer array is transcribed into a long precursor crRNA, which is further processed to liberate mature crRNAs that each specifies a single target. During interference, crRNAs combine with one or more Cas proteins to form an effector complex, which recognizes and degrades nucleic acids (called “protospacers”) that are complementary to the crRNA. crRNA biogenesis and interference together constitute the defense phase of CRISPR–Cas immunity. Although all known CRISPR–Cas systems adhere to this general pathway, they exhibit striking phylogenetic and mechanistic diversity. The current classification scheme places these systems into two broad classes, six distinct Types (I–VI), and dozens of subtypes based upon *cas* gene composition and differences in detailed mechanisms [42,43]. According to this scheme, Class 1 systems (Types I, III, and IV) encode multisubunit effector complexes, while Class 2 systems (Types II, V, and VI) rely upon a single subunit to destroy nucleic acid invaders.

Recent reports have shown that diverse CRISPR–Cas systems (Types I, II, and III) can be used as powerful tools to facilitate phage genome engineering [28,33,34,35,36,37]. In these studies, representative members of all three families of tailed phages (*Myoviridae*, *Siphoviridae*, and *Podoviridae*) were successfully edited by applying variations of the same basic approach, in which CRISPR–Cas defense is used as a mechanism to counterselect for phages that have recombined with a “donor DNA” construct supplied *in trans* (Figure 1B). The donor DNA contains a segment of the phage genome that bears the desired mutations within the protospacer region flanked by sequences homologous to the phage genome on both sides. Phages that recombine with the construct and acquire the mutations can, thus, escape CRISPR immunity and complete their replication cycle. Although recombination rates may be very low, immunity against wild-type phages effectively enriches for the rare recombinants. The CRISPR–Cas systems used in this approach and phage editing efficiencies are summarized in Table 1. The detailed defense mechanisms of each CRISPR–Cas type, specific strategies used, and phage editing outcomes are described in the sections below.

### 3.1. Type I CRISPR–Cas Systems (CRISPR–Cas3)

**Figure 2:**
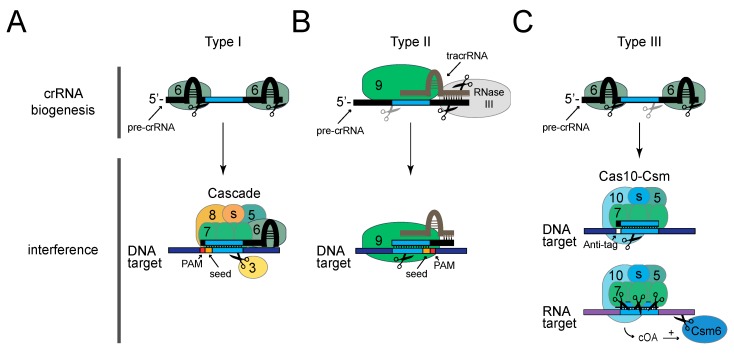
The three main CRISPR–Cas Types that have been successfully used to edit phages. Shown are protein and nucleic acid requirements for crRNA biogenesis and interference steps in representative CRISPR–Cas systems: Type I-E from *E. coli* (**A**), Type II-A from *S. pyogenes* (**B**), and Type III-A from *S. epidermidis* (**C**). Numbered ovals represent corresponding Cas proteins, and “s” represents one or more copies of a small subunit specific to each subtype. Black scissors represent cleavage points made by the overlapping protein subunit, while grey scissors represent cleavage events catalyzed by non-Cas and/or unknown nucleases. For Type I and II systems, the PAM and seed sequences are represented by red and orange rectangles, respectively. For Type III systems, the crRNA tag is represented by a black square, and the opposing anti-tag is shown as a white square. cOA, cyclic oligoadenylates.

The best-characterized example of a Type I CRISPR–Cas system is the model Type I-E system found in *E. coli* (Figure 2A) [41,44,45,46,47,48]. In this system, crRNA biogenesis relies upon the activity of the endoribonuclease Cas6 (also called CasE or Cas6e), which recognizes and cuts the precursor crRNA within each repeat. Cas6 and the crRNA are joined by the large type-specific subunit Cas8 (also called CasA or Cse1), two copies of a small subtype-specific subunit (CasB or Cse2 in *E. coli*), six copies of Cas7 (also called CasC), and Cas5 (also called CasD). The resulting ribonucleoprotein complex, known as Cascade (CRISPR-associated complex for antiviral defense), is capable of detecting invading nucleic acids that harbor base pair complementarity with the crRNA. An additional sequence requirement for interference is the presence of a two- to six- nucleotide protospacer-adjacent motif (PAM) located on the non-complementary DNA strand [49,50]. Once detected by Cascade, the DNA target is cleaved by an additional protein, the helicase-nuclease Cas3 [41,44,45]. As one important consideration for phage engineering, Type I systems do not constitute an impenetrable barrier to phage infection, as phages that randomly acquire point mutations within the PAM or the first 6–8 nucleotides in the protospacer (called the “seed”) [51,52] can escape CRISPR interference altogether. These natural phage “escapers” can proliferate and obscure the desired recombinants; however, they present only a minor impediment to the recovery of desired mutants according to two recent reports that demonstrate the utility of Type I-E systems in editing phages that infect *E. coli* and *Vibrio cholerae* [33,34].

In *E. coli*, non-essential genes were deleted from the lytic *Podoviridae* phage T7 using a two-step approach [33]. In the first step, phages were propagated on a strain harboring a plasmid containing the donor DNA, which consisted of 120 nucleotides of phage-derived sequence flanking the gene to be deleted. Following propagation on this strain, phage progeny consisted of a mixture of wild-type (the vast majority) and those that had recombined with the plasmid, and thus, had lost the gene of interest. In the second step, the rare phage recombinants were enriched by plating the mixture of phages on a “targeting strain” bearing three plasmids that encode *cascade*, *cas3*, and spacer sequences complementary to the gene of interest. As expected, the phages recovered following the second step consisted of a mixture of at least two types of mutants: (i) phages that naturally escaped CRISPR interference through the incorporation of random mutations in the protospacer region, and (ii) phages that had recombined with the plasmid in the first step, and thus had acquired the desired deletion. Two genes were deleted independently using this method, and it was found that 38% and 42% of recovered phages, respectively, fell into the latter category. For a detailed protocol of this method, the reader is referred to [53].

Another study used the same underlying principle in a one-step approach to delete and replace genes in the *V. cholerae* phage ICP1_2011_A, a lytic phage belonging to the family *Myoviridae* [34]. Here, a single plasmid containing a targeting spacer and the donor DNA construct with desired mutations (flanked by ~250 nucleotides of homology on each side) were introduced into a *V. cholerae* strain bearing a Type I-E CRISPR–Cas system in its genome. During a single round of phage infection on this strain, targeting and editing occur simultaneously. Three independent mutations were made using this system: a small deletion (33 nt) encompassing precisely the protospacer region, a large in-frame deletion of two adjacent genes (>2500 nucleotides in length), and replacement of the latter two genes with the gene for green fluorescent protein (>700 nucleotides in length). While the small deletion was recovered in 100% of the mutant phages tested (8/8), the large deletion and gene replacement presumably occurred at lower frequencies, with the desired mutation found in ~50% of recovered phages (7/12 and 4/8, respectively).

### 3.2. Type II CRISPR–Cas Systems (CRISPR–Cas9)

Falling within the broad category of Class 2 systems, Type II CRISPR–Cas systems use a single multidomain protein, Cas9, to carry out defense (Figure 2B). Among the most simple of the CRISPR–Cas systems [54], Type II (also known as CRISPR–Cas9) are, thus far, the most commonly used for genome editing applications [19]. Type II-A systems originating from *Streptococcus pyogenes* and *Streptococcus thermophilus* have been used to edit phages that infect diverse hosts. In these systems, crRNA biogenesis relies upon a small trans-activating crRNA (tracrRNA), which contains a region complementary to repeat-derived sequences in the crRNA. Base pairing between the tracrRNA and precursor crRNA facilitates the cleavage of both RNAs by the host-encoded nuclease RNase III [55], an event that defines the 3’-end of the crRNA. A second processing step by an unknown nuclease trims the 5’-end to generate mature crRNAs. While bound to both processed small RNAs, Cas9 can sense and cleave double-stranded DNA targets using its two independent active sites [55,56]. Similarly to Type I, Type II CRISPR–Cas systems require the presence of a PAM [49,57] and perfect complementarity between the crRNA and protospacer in a seed region [58] to license interference.

Although phage escape mutants that have randomly acquired point mutations in the PAM or seed may obscure phage recombinants when specific edits are desired, escapers containing deletions in a specific gene of interest can be enriched by targeting the gene in the absence of a donor DNA. Just such an approach was used to identify nonessential genes in the lytic *Siphoviridae* phage 2972 [28]. In this study, four different *S. thermophilus* isolates, each bearing a Type II-A CRISPR–Cas system with a natural spacer targeting one of four phage genes with unknown functions, were used to generate escape mutants. The expectation is that if a targeted gene is not essential, phage escapers harboring random deletions or nonsense mutations in the gene could be readily isolated, while the inability to find such inactivating mutations in natural escapers might indicate the gene is essential for phage survival. Of the four genes targeted, it was found that three could bear deletions and nonsense mutations, suggesting the latter are dispensable for phage survival. Although this approach requires tedious screening efforts to (i) identify a CRISPR locus that already contains the desired spacer and (ii) isolate escaper phages with the desired mutations, this strategy could be useful in non-model organisms that are refractory to transformation with foreign DNA. A plasmid-based system was also developed in *S. thermophilus* to make precise edits, including point mutations, deletions, and a gene replacement using a donor DNA construct. Interestingly, natural phage escapers did not appear to outnumber the desired recombinants, likely owing to high recombination frequencies in this system [28].

CRISPR–Cas9 has also been used in heterologous hosts to engineer lytic phages. In one study, a plasmid bearing the Type II-A system from *S. pyogenes* was introduced into *Lactococcus lactis* and used in conjunction with a separate plasmid-encoded donor DNA to engineer the lytic *Siphoviridae* phage p2 [35]. Interestingly, when programmed with a spacer that targets the phage, the CRISPR–Cas system on its own provided only modest protection, reducing plaque counts by 1/3–1/2; however, in the presence of the donor DNA, the desired mutants could be recovered with apparent ease following multiple rounds of phage propagation on the strain containing both the targeting spacer and the donor DNA. This system was used to introduce deletions, insertions, and point mutations in several genetic loci. In another study, the same Type II-A system was used to make similar mutations in the *E. coli* phage T4 [37], a lytic phage belonging to the family *Myoviridae*. In this report, the effect of phage genome modifications was investigated, as phage T4 is known to harbor glucosyl-hydroxymethylcytosine modifications that are resistant to the activity of restriction endonucleases [22]. In order to determine the effect of these modifications on CRISPR–Cas9 defense, spacers targeting 25 genetic loci across the T4 genome were designed, and immunity was tested against both wild-type T4 and a mutant lacking the modifications. It was found that although wild-type phages appeared less susceptible to CRISPR–Cas9 interference, this system could still be harnessed to counterselect for recombinant phages that have acquired mutations in the desired gene(s). Of note, this study showed that nonsense mutations could be introduced into essential phage genes using an *E. coli* amber suppressor strain, which allows readthrough of the stop codon and proliferation of the phage mutant on this strain. A similar approach using suppressor strains in conjunction with CRISPR–Cas editing could be feasibly applied in a broad range of hosts to study the functions of essential phage genes.

### 3.3. Type III CRISPR–Cas Systems (CRISPR–Cas10)

Type III systems are the most elaborate of the three main CRISPR–Cas types. One of the best characterized examples is the Type III-A system found in *Staphylococcus epidermidis* (Figure 2C) [59,60]. Similarly to Type I, crRNA processing in this system relies on the Cas6 endonuclease, which defines the 5′-ends [61,62]; however, additional cleavage by non-Cas cellular nucleases are required for crRNA maturation on their 3′-ends [63]. Mature crRNAs are bound to a multi-subunit effector complex known as Cas10-Csm, composed of the large type-specific subunit Cas10 (also called Csm1), a small subtype-specific subunit (Csm2 in *S. epidermidis*), Cas5 (also called Csm4), and multiple copies of Cas7 homologs (Csm3 and Csm5) [64]. This complex, in conjunction with Csm6 can recognize and destroy foreign nucleic acids (both DNA and RNA) in a transcription-dependent manner [64,65,66]. Within Cas10–Csm, the binding of the crRNA to a complementary RNA transcript triggers interference through the activity of at least three distinct nucleases: Cas10 cleaves the non-template (coding) DNA strand, each Csm3 subunit slices RNA within the protospacer region, and Csm6 processively degrades targeted transcripts [67]. An additional layer of complexity was recently discovered in Type III systems, in which Cas10 generates cyclic oligoadenylate molecules that bind and stimulate Csm6 [68,69]. Although a PAM has not been detected for Type III systems, the absence of complementarity between the eight nucleotides on the 5′-end of crRNAs (called the tag) and the opposing region adjacent to the protospacer (called the anti-tag) is required to license interference [60]. In addition, a defined seed sequence seems to be altogether absent [70], and the crRNA–protospacer pair can tolerate up to five mismatches and still carry out interference [71]. One important consideration is that interference in Type III systems occurs only when the targeted locus is actively transcribed, therefore, these systems are not suitable for engineering late genes in lysogenic phages, which remain silent during lysogeny. However, this issue is not expected to pose a problem for the genetic engineering of lytic phages.

The robust immunity against lytic phages exhibited by Type III systems make them particularly suited to phage engineering applications, as was demonstrated recently in *S. epidermidis* [36]. In this study, point mutations in multiple genetic loci were introduced into two lytic phages, *Podoviridae* phage Andhra [72] and *Myoviridae* phage ISP [73], using the chromosome-encoded Type III-A system and a single plasmid containing the targeting spacer and donor DNA. Spacers targeting multiple genetic loci across both phage genomes yielded zero phage escapers, suggesting this system would make an exceptional counterselection mechanism for phage recombinants. Indeed, following propagation on “editing strains” that contain both the targeting spacer and donor DNA construct, 100% of phages selected (20/20 for each gene edited) had acquired the desired edits. A plasmid bearing the entire Type III-A system was also shown to work as efficiently when introduced into *S. aureus*, a heterologous CRISPR-less host. Additionally, a modified approach was used to introduce mutations up to ~500 nucleotides distal to the targeted protospacer. Due to the unique targeting requirements for Type III systems, the development of a bioinformatics tool allowed the identification of potential protospacers across phage genomes, which were found to be enriched in the phages tested (~12 possible protospacers per 100 nucleotides of coding sequence).

## 4. Conclusions and Future Perspectives

It is fortunate that along with the rich treasure chest of phage genetic diversity, nature has also provided the keys, a suite of anti-phage defense systems, which offer the means to unlock the secrets of phage genes of unknown functions. CRISPR–Cas systems are widespread in the prokaryotic world, with some organisms harboring more than one Type [38], thus offering multiple options. The reports described herein amply demonstrate that CRISPR–Cas systems in diverse organisms can be harnessed to accelerate phage evolution in a controlled environment in order to introduce specific, desired mutations. Representative tailed phages from all three families of the *Caudovirales* order have been genetically engineered using this approach. As with any genome editing tool, one potential issue that could arise is the introduction of unintended mutations through off-target cleavage by CRISPR-associated nucleases. However, such off-target effects can be easily ruled out by whole phage sequencing once the desired mutants are recovered. As another potential setback, phages that encode CRISPR–Cas inhibitors, which have been described for Types I and II systems [74,75,76], might undermine these efforts. If one such phage is encountered, finding the right CRISPR–Cas type for the job might be challenging, but not impossible, as CRISPR–Cas systems are diverse, relatively widespread, and functional in heterologous hosts. Even phages with RNA genomes can be feasibly accessed and engineered using CRISPR–Cas systems that target RNA (such as Types III and VI), and phages that infect archaea are, at least in theory, accessible with the CRISPR–Cas systems that abound in these organisms (such as Types I and III). It is therefore plausible to envision that nature’s suite of CRISPR–Cas systems will soon become indispensable tools in phage research that will significantly advance our basic understanding of phage biology and accelerate the development of novel technologies based on these pervasive organisms.

## Figures and Tables

**Table 1:** Summary of CRISPR–Cas systems used to edit phages and corresponding efficiencies *^a^*.

CRISPR Type	Host Organism	Phages Edited *^b^*	Mutations Introduced	Editing Efficiency *^c^* (# Desired Mutants/Total # Phages Screened)	Ref.
I-E	*E. coli*	T7 (P)	two single gene deletions	38% (17/44) and 42% (15/36)	[33]
I-E	*V. cholerae*	ICP1_2011_A (M)	33 nt deletion deletion of two genes gene exchange	100% (8/8) 58% (7/12) 50% (4/8)	[34]
II-A	*S. thermophilus*	2972 (S)	point mutation 2 nt deletion single gene deletion gene exchange	100% (10/10) 80% (8/10) 100% (10/10) ND *^d^*	[28]
II-A	*L. lactis*	p2 (S)	single gene deletion point mutation 18 nt insertion	ND ND ND	[35]
II-A	*E. coli*	T4 (M)	point mutations single gene deletion	100% (20/20) 100% (5/5)	[37]
III-A	*S. epidermidis* *S. aureus*	Andhra (P) ISP (M)	silent mutations in multiple genetic loci	100% (20/20) at all loci tested	[36]

*^a^* Data is shown only in cases where a donor DNA construct was used in conjunction with CRISPR–Cas immunity to introduce specific edits. *^b^* (P), (S), and (M) refer to phage families *Podoviridae*, *Siphoviridae*, and *Myoviridae*, respectively. *^c^* Editing efficiency refers to the fraction of phages selected that had acquired the desired mutations as determined by PCR, restriction digest, and/or DNA sequencing; *^d^* ND, not determined.

## Supplementary Materials

The following are available online at http://www.mdpi.com/1999-4915/10/6/335/s1, Table S1: Characteristics of complete phage genomes listed in the NCBI database.

## References

[1] Hatfull G.F. (2015). Dark Matter of the Biosphere: The Amazing World of Bacteriophage Diversity. J. Virol..

[2] Brüssow H., Hendrix R.W. (2002). Phage Genomics: Small Is Beautiful. Cell.

[3] Comeau M., Hatfull G.F., Krisch H.M., Lindell D., Mann N.H., Prangishvili D. (2008). Exploring the Prokaryotic Virosphere. Res. Microbiol..

[4] Bergh O., Børsheim K.Y., Bratbak G., Heldal M. (1989). High Abundance of Viruses Found in Aquatic Environments. Nature.

[5] Breitbart M., Rohwer F. (2005). Here a Virus, There a Virus, Everywhere the Same Virus?. Trends Microbiol..

[6] Brüssow H., Canchaya C., Hardt W., Bru H. (2004). Phages and the Evolution of Bacterial Pathogens: From Genomic Rearrangements to Lysogenic Conversion. Microbiol. Mol. Biol. Rev..

[7] Waldor M.K., Mekalanos J.J. (1996). Lysogenic Conversion by a Filamentous Phage Encoding Cholera Toxin. Science.

[8] Zinder N.D., Lederberg J. (1952). Genetic Exchange in *Salmonella*. J. Bacteriol..

[9] Suttle C.A. (2007). Marine Viruses—Major Players in the Global Ecosystem. Nat. Rev. Microbiol..

[10] Reche I., D’Orta G., Mladenov N., Winget D.M., Suttle C.A. (2018). Deposition Rates of Viruses and Bacteria above the Atmospheric Boundary Layer. ISME J..

[11] Van Wezenbeek P.M., Hulsebos T.J., Schoenmakers J.G. (1980). Nucleotide Sequence of the Filamentous Bacteriophage M13 DNA Genome: Comparison with Phage Fd. Gene.

[12] Krisch H.M., Comeau A.M. (2008). The Immense Journey of Bacteriophage T4-From d’Hérelle to Delbrück and Then to Darwin and beyond. Res. Microbiol..

[13] Casjens S.R., Hendrix R.W. (2015). Bacteriophage Lambda: Early Pioneer and Still Relevant. Virology.

[14] Salmond G.P.C., Fineran P.C. (2015). A Century of the Phage: Past, Present and Future. Nat. Rev. Microbiol..

[15] Hershey A.D., Chase M. (1952). Independent Functions of Viral Protein and Nucleic Acid in Growth of Bacteriophage. J. Gen. Physiol..

[16] Brenner S., Jacob F., Meselson M. (1961). An Unstable Intermediate Carrying Information from Genes to Ribosomes for Protein Synthesis. Nature.

[17] Crick F.H.C., Barnett L., Brenner S., Watts-Tobin R.J. (1961). General Nature of the Genetic Code for Proteins. Nature.

[18] Barrangou R., Fremaux C., Deveau H., Richards M., Boyaval P., Moineau S., Romero D.A., Horvath P. (2007). CRISPR Provides Acquired Resistance against Viruses in Prokaryotes. Science.

[19] Doudna J.A., Charpentier E. (2014). The New Frontier of Genome Engineering with CRISPR-Cas9. Science.

[20] Twort F.W. (1915). An Investigation on the Nature of Ultra-Microscopic Viruses. Lancet.

[21] D’Herelle F. (1917). Sur Un Microbe Invisible Antagoniste Des Bacilles Dysentériques. CR Acad. Sci. Paris.

[22] Miller E.S., Kutter E., Mosig G., Arisaka F., Kunisawa T., Rüger W. (2003). Bacteriophage T4 Genome. Microbiol. Mol. Biol. Rev..

[23] Pope W.H., Bowman C.A., Russell D.A., Jacobs-Sera D., Asai D.J., Cresawn S.G., Jacobs W.R., Hendrix R.W., Lawrence J.G., Hatfull G.F. (2015). Whole Genome Comparison of a Large Collection of Mycobacteriophages Reveals a Continuum of Phage Genetic Diversity. eLife.

[24] Henn M.R., Sullivan M.B., Stange-Thomann N., Osburne M.S., Berlin A.M., Kelly L., Yandava C., Kodira C., Zeng Q., Weiand M. (2010). Analysis of High-Throughput Sequencing and Annotation Strategies for Phage Genomes. PLoS ONE.

[25] Rihtman B., Meaden S., Clokie M.R.J., Koskella B., Millard A.D. (2016). Assessing Illumina Technology for the High-Throughput Sequencing of Bacteriophage Genomes. PeerJ.

[26] Pires D.P., Cleto S., Sillankorva S., Azeredo J., Lu T.K. (2016). Genetically Engineered Phages: A Review of Advances over the Last Decade. Microbiol. Mol. Biol. Rev..

[27] Loessner M.J., Rees C.E., Stewart G.S., Scherer S. (1996). Construction of Luciferase Reporter Bacteriophage A511::luxAB for Rapid and Sensitive Detection of Viable *Listeria* Cells. Appl. Environ. Microbiol..

[28] Martel B., Moineau S. (2014). CRISPR-Cas: An Efficient Tool for Genome Engineering of Virulent Bacteriophages. Nucleic Acids Res..

[29] Chan L.Y., Kosuri S., Endy D. (2005). Refactoring Bacteriophage T7. Mol. Syst. Biol..

[30] Marinelli L.J., Piuri M., Swigoňová Z., Balachandran A., Oldfield L.M., van Kessel J.C., Hatfull G.F. (2008). BRED: A Simple and Powerful Tool for Constructing Mutant and Recombinant Bacteriophage Genomes. PLoS ONE.

[31] Ando H., Lemire S., Pires D.P., Lu T.K. (2015). Engineering Modular Viral Scaffolds for Targeted Bacterial Population Editing. Cell Syst..

[32] Kilcher S., Studer P., Muessner C., Klumpp J., Loessner M.J. (2018). Cross-Genus Rebooting of Custom-Made, Synthetic Bacteriophage Genomes in L-Form Bacteria. Proc. Natl. Acad. Sci. USA.

[33] Kiro R., Shitrit D., Qimron U. (2014). Efficient Engineering of a Bacteriophage Genome Using the Type I-E CRISPR-Cas System. RNA Biol..

[34] Box A.M., McGuffie M.J., O’Hara B.J., Seed K.D. (2016). Functional Analysis of Bacteriophage Immunity through a Type I-E CRISPR-Cas System in *Vibrio cholerae* and Its Application in Bacteriophage Genome Engineering. J. Bacteriol..

[35] Lemay M.-L., Tremblay D.M., Moineau S. (2017). Genome Engineering of Virulent Lactococcal Phages Using CRISPR-Cas9. ACS Synth. Biol..

[36] Bari S.M.N., Walker F.C., Cater K., Aslan B., Hatoum-Aslan A. (2017). Strategies for Editing Virulent Staphylococcal Phages Using CRISPR-Cas10. ACS Synth. Biol..

[37] Tao P., Wu X., Tang W.-C., Zhu J., Rao V. (2017). Engineering of Bacteriophage T4 Genome Using CRISPR-Cas9. ACS Synth. Biol..

[38] Grissa I., Vergnaud G., Pourcel C. (2007). The CRISPRdb Database and Tools to Display CRISPRs and to Generate Dictionaries of Spacers and Repeats. BMC Bioinform..

[39] Godde J.S., Bickerton A. (2006). The Repetitive DNA Elements Called CRISPRs and Their Associated Genes: Evidence of Horizontal Transfer among Prokaryotes. J. Mol. Evol..

[40] Haft D.H., Selengut J., Mongodin E.F., Nelson K.E. (2005). A Guild of 45 CRISPR-Associated (Cas) Protein Families and Multiple CRISPR/Cas Subtypes Exist in Prokaryotic Genomes. PLoS Comput. Biol..

[41] Brouns S.J.J., Jore M.M., Lundgren M., Westra E.R., Slijkhuis R.J.H., Snijders A.P.L., Dickman M.J., Makarova K.S., Koonin E.V., van der Oost J. (2008). Small CRISPR RNAs Guide Antiviral Defense in Prokaryotes. Science.

[42] Makarova K.S., Wolf Y.I., Alkhnbashi O.S., Costa F., Shah S.A., Saunders S.J., Barrangou R., Brouns S.J.J., Charpentier E., Haft D.H. (2015). An Updated Evolutionary Classification of CRISPR-Cas Systems. Nat. Rev. Microbiol..

[43] Koonin E.V., Makarova K.S., Zhang F. (2017). Diversity, Classification and Evolution of CRISPR-Cas Systems. Curr. Opin. Microbiol..

[44] Sinkunas T., Gasiunas G., Fremaux C., Barrangou R., Horvath P., Siksnys V. (2011). Cas3 Is a Single-Stranded DNA Nuclease and ATP-Dependent Helicase in the CRISPR/Cas Immune System. EMBO J..

[45] Beloglazova N., Petit P., Flick R., Brown G., Savchenko A., Yakunin A.F. (2011). Structure and Activity of the Cas3 HD Nuclease MJ0384, an Effector Enzyme of the CRISPR Interference. EMBO J..

[46] Westra E.R., van Erp P.B.G., Künne T., Wong S.P., Staals R.H.J., Seegers C.L.C., Bollen S., Jore M.M., Semenova E., Severinov K. (2012). CRISPR Immunity Relies on the Consecutive Binding and Degradation of Negatively Supercoiled Invader DNA by Cascade and Cas3. Mol. Cell.

[47] Jackson R.N., Golden S.M., van Erp P.B.G., Carter J., Westra E.R., Brouns S.J.J., van der Oost J., Terwilliger T.C., Read R.J., Wiedenheft B. (2014). Crystal Structure of the CRISPR RNA–Guided Surveillance Complex from *Escherichia coli*. Science.

[48] Mulepati S., Héroux A., Bailey S. (2014). Crystal Structure of a CRISPR RNA–Guided Surveillance Complex Bound to a ssDNA Target. Science.

[49] Deveau H., Barrangou R., Garneau J.E., Labonté J., Fremaux C., Boyaval P., Romero D.A., Horvath P., Moineau S. (2008). Phage Response to CRISPR-Encoded Resistance in *Streptococcus thermophilus*. J. Bacteriol..

[50] Mojica F.J.M., Díez-Villaseñor C., García-Martínez J., Almendros C. (2009). Short Motif Sequences Determine the Targets of the Prokaryotic CRISPR Defence System. Microbiology.

[51] Semenova E., Jore M.M., Datsenko K.A., Semenova A., Westra E.R., Wanner B., van der Oost J., Brouns S.J.J., Severinov K. (2011). Interference by Clustered Regularly Interspaced Short Palindromic Repeat (CRISPR) RNA Is Governed by a Seed Sequence. Proc. Natl. Acad. Sci. USA.

[52] Wiedenheft B., van Duijn E., Bultema J.B., Waghmare S., Zhou K., Barendregt A., Westphal W., Heck A.J.R., Boekema E.J., Dickman M.J. (2011). RNA-Guided Complex from a Bacterial Immune System Enhances Target Recognition through Seed Sequence Interactions. Proc. Natl. Acad. Sci. USA.

[53] Manor M., Qimron U. (2017). Selection of Genetically Modified Bacteriophages Using the CRISPR-Cas System. Bio-Protocol.

[54] Chylinski K., Makarova K.S., Charpentier E., Koonin E.V. (2014). Classification and Evolution of Type II CRISPR-Cas Systems. Nucleic Acids Res..

[55] Deltcheva E., Chylinski K., Sharma C.M., Gonzales K., Chao Y., Pirzada Z.A., Eckert M.R., Vogel J., Charpentier E. (2011). CRISPR RNA Maturation by Trans-Encoded Small RNA and Host Factor RNase III. Nature.

[56] Garneau J.E., Dupuis M.È., Villion M., Romero D.A., Barrangou R., Boyaval P., Fremaux C., Horvath P., Magadán A.H., Moineau S. (2010). The CRISPR/cas Bacterial Immune System Cleaves Bacteriophage and Plasmid DNA. Nature.

[57] Sapranauskas R., Gasiunas G., Fremaux C., Barrangou R., Horvath P., Siksnys V. (2011). The *Streptococcus thermophilus* CRISPR/Cas System Provides Immunity in *Escherichia Coli*. Nucleic Acids Res..

[58] Jiang W., Bikard D., Cox D., Zhang F., Marraffini L.A. (2013). CRISPR-Assisted Editing of Bacterial Genomes. Nat. Biotechnol..

[59] Marraffini L.A., Sontheimer E.J. (2008). CRISPR Interference Limits Horizontal Gene Transfer in Staphylococci by Targeting DNA. Science.

[60] Marraffini L.A., Sontheimer E.J. (2010). Self vs. Non-Self Discrimination during CRISPR RNA-Directed Immunity. Nature.

[61] Hatoum-Aslan A., Maniv I., Marraffini L.A. (2011). Mature Clustered, Regularly Interspaced, Short Palindromic Repeats RNA (crRNA) Length Is Measured by a Ruler Mechanism Anchored at the Precursor Processing Site. Proc. Natl. Acad. Sci. USA.

[62] Hatoum-Aslan A., Maniv I., Samai P., Marraffini L.A. (2014). Genetic Characterization of Antiplasmid Immunity through a Type III-A CRISPR-Cas System. J. Bacteriol..

[63] Walker F.C., Chou-Zheng L., Dunkle J.A., Hatoum-Aslan A. (2017). Molecular Determinants for CRISPR RNA Maturation in the Cas10—Csm Complex and Roles for Non-Cas Nucleases. Nucleic Acids Res..

[64] Hatoum-Aslan A., Samai P., Maniv I., Jiang W., Marraffini L.A. (2013). A Ruler Protein in a Complex for Antiviral Defense Determines the Length of Small Interfering CRISPR RNAs. J. Biol. Chem..

[65] Goldberg G.W., Jiang W., Bikard D., Marraffini L.A. (2014). Conditional Tolerance of Temperate Phages via Transcription-Dependent CRISPR-Cas Targeting. Nature.

[66] Samai P., Pyenson N., Jiang W., Goldberg G.W., Hatoum-Aslan A., Marraffini L.A. (2015). Co-Transcriptional DNA and RNA Cleavage during Type III CRISPR-Cas Immunity. Cell.

[67] Jiang W., Samai P., Marraffini L.A. (2016). Degradation of Phage Transcripts by CRISPR-Associated RNases Enables Type III CRISPR-Cas Immunity. Cell.

[68] Kazlauskiene M., Kostiuk G., Venclovas Č., Tamulaitis G., Siksnys V. (2017). A Cyclic Oligonucleotide Signaling Pathway in Type III CRISPR-Cas Systems. Science.

[69] Niewoehner O., Garcia-Doval C., Rostøl J.T., Berk C., Schwede F., Bigler L., Hall J., Marraffini L.A., Jinek M. (2017). Type III CRISPR–Cas Systems Produce Cyclic Oligoadenylate Second Messengers. Nature.

[70] Maniv I., Jiang W., Bikard D., Marraffini L.A. (2016). Impact of Different Target Sequences on Type III CRISPR-Cas Immunity. J. Bacteriol..

[71] Pyenson N.C., Gayvert K., Varble A., Elemento O., Marraffini L.A. (2017). Broad Targeting Specificity during Bacterial Type III CRISPR-Cas Immunity Constrains Viral Escape. Cell Host Microbe.

[72] Cater K., Dandu V.S., Bari S.M.N., Lackey K., Everett G.F.K., Hatoum-Aslan A. (2017). A Novel *Staphylococcus* Podophage Encodes a Unique Lysin with Unusual Modular Design. mSphere.

[73] Vandersteegen K., Mattheus W., Ceyssens P.J., Bilocq F., de Vos D., Pirnay J.P., Noben J.P., Merabishvili M., Lipinska U., Hermans K. (2011). Microbiological and Molecular Assessment of Bacteriophage ISP for the Control of *Staphylococcus aureus*. PLoS ONE.

[74] Bondy-Denomy J., Pawluk A., Maxwell K.L., Davidson A.R. (2013). Bacteriophage Genes that Inactivate the CRISPR/Cas Bacterial Immune System. Nature.

[75] Pawluk A., Bondy-Denomy J., Cheung V.H.W., Maxwell K.L., Davidson R. (2014). A New Group of Phage Anti-CRISPR Genes Inhibits the Type I-E CRISPR-Cas System of *Pseudomonas aeruginosa*. mBio.

[76] Pawluk A., Amrani N., Zhang Y., Garcia B., Hidalgo-Reyes Y., Lee J., Edraki A., Shah M., Sontheimer E.J., Maxwell K.L. (2016). Naturally Occurring Off-Switches for CRISPR-Cas9. Cell.

